# When a Contaminant Is Not a Contaminant: Corynebacterium striatum Causing Native Tricuspid Valve Endocarditis

**DOI:** 10.51894/001c.165581

**Published:** 2026-07-31

**Authors:** Venkata Anirudh Chunchu, Hemanth Kumar Kattula, Krishna Teja Challa, Berger Gregory

**Affiliations:** 1 DMC - Sinai Grace

## 73

### Introduction

Corynebacterium striatum is commonly regarded as a skin contaminant when isolated from blood cultures. However, increasing evidence suggests it is an emerging opportunistic pathogen capable of causing invasive infections, including infective endocarditis. We report a case of catheter-associated native tricuspid valve infective endocarditis caused by Corynebacterium striatum in a patient receiving hemodialysis.

### Case Description

An 80-year-old man with end-stage renal disease on hemodialysis via a right internal jugular tunneled catheter, Alzheimer’s dementia, prior cerebrovascular accident, heart failure with preserved ejection fraction, peripheral arterial disease status post left below-knee amputation, diabetes mellitus, chronic anemia, and chronic obstructive pulmonary disease presented from a skilled nursing facility with fever, hypotension, tachycardia, and altered mental status. On presentation, he was febrile (38.4 °C), hypotensive (90s/60s mmHg), and tachycardic. Laboratory studies showed leukocytosis (WBC 17.5 × 10⁹/L), blood urea nitrogen 114 mg/dL, and creatinine 7.57 mg/dL. Physical exam revealed somnolence and coarse breath sounds. The dialysis catheter site appeared clean. Empiric vancomycin, cefepime, and metronidazole were initiated for suspected sepsis. The patient developed persistent hypotension requiring vasopressors and ICU admission. Blood cultures from two sets grew gram-positive bacilli later identified as Corynebacterium striatum. Transthoracic echocardiography suggested tricuspid vegetation, and transesophageal echocardiography confirmed large mobile vegetations involving all three tricuspid leaflets, mild-to-moderate tricuspid regurgitation and a non-occlusive thrombus in the superior vena cava extending along the dialysis catheter. Antibiotics were narrowed to intravenous vancomycin per Infectious Disease recommendations. Cardiothoracic surgery deemed the patient a poor surgical candidate due to severe comorbidities. The tunneled dialysis catheter was removed, repeat blood cultures cleared. The patient was discharged to a long-term care facility to complete a four-week course of intravenous vancomycin.

### Discussion

Corynebacterium striatum, historically regarded as a blood culture contaminant, is increasingly recognized as a clinically significant pathogen in patients with intravascular devices. Hemodialysis catheters can serve as portals of entry, predisposing patients to right-sided infective endocarditis. Isolation of this organism from multiple blood cultures should prompt careful clinical evaluation rather than dismissal as contamination. Early echocardiographic assessment, removal of infected intravascular hardware, and targeted antimicrobial therapy are essential for effective management and improved outcomes.

